# 3D versus 2D Systematic Transrectal Ultrasound-Guided Prostate Biopsy: Higher Cancer Detection Rate in Clinical Practice

**DOI:** 10.1155/2013/783243

**Published:** 2013-11-17

**Authors:** Alexandre Peltier, Fouad Aoun, Fouad El-Khoury, Eric Hawaux, Ksenija Limani, Krishna Narahari, Nicolas Sirtaine, Roland van Velthoven

**Affiliations:** ^1^Department of Urology, Jules Bordet Institute, 1 Rue Héger-Bordet, 1000 Brussels, Belgium; ^2^Université Libre de Bruxelles, 50 Franklin Roosevelt Avenue, 1050 Brussels, Belgium; ^3^Department of Anatomopathology, Jules Bordet Institute, 1 Rue Héger-Bordet, 1000 Brussels, Belgium

## Abstract

*Objectives*. To compare prostate cancer detection rates of extended 2D versus 3D biopsies and to further assess the clinical impact of this method in day-to-day practice. *Methods*. We analyzed the data of a cohort of 220 consecutive patients with no prior history of prostate cancer who underwent an initial prostate biopsy in daily practice due to an abnormal PSA and/or DRE using, respectively, the classical 2D and the new 3D systems. All the biopsies were done by a single experienced operator using the same standardized protocol. *Results*. There was no significant difference in terms of age, total PSA, or prostate volume between the two groups. However, cancer detection rate was significantly higher using the 3D versus the 2D system, 50% versus 34% (*P* < 0.05). There was no statistically significant difference while comparing the 2 groups in term of nonsignificant cancer detection. *Conclusion*. There is reasonable evidence demonstrating the superiority of the 3D-guided biopsies in detecting prostate cancers that would have been missed using the 2D extended protocol.

## 1. Introduction

Prostate cancer (PC) is the most common cancer in elderly men and the second most common cause of cancer death in the western world [1, 2]. Grey scale (GS) two dimension (2D) transrectal ultrasound- (TRUS-) guided systematic prostate biopsy sampling is the clinical standard for PC diagnosis [3]. In clinical practice, PC detection rate of GS 2D TRUS-guided needle biopsies is only 30–40% in initial prostate biopsy [4–7] in a screened population and 30% to 50% of PC that require definitive treatment remain undetected [8, 9]. Furthermore, prostate remains the only organ where biopsy is a blind uniform sampling technique due to the poor visibility of cancer in GS 2D TRUS images and the limited anatomical context to guide needles to suspicious locations in the 2D TRUS plane [10]. Recent developments in systems and imaging modalities have led to a promising advance in mapping and correctly tracking target regions. In the last years, the GS three dimension (3D) TRUS-guided biopsy has been introduced as a new technique that improves prostate sampling as well as clinical quality management [11]. Based on these findings, we compare a consecutive series of 220 patients who underwent a prostate biopsy in order to assess the impact of 3D versus 2D TRUS-guided systematic prostate biopsy on the detection of PC and its clinical effect in a routine day-to-day practice. 

## 2. Materials and Methods

The study involved a cohort of 220 consecutive patients, with no prior history of prostate cancer, who underwent prostate biopsy due to an abnormal PSA and/or DRE in Urology Department at Jules Bordet Institute between January 2009 and August 2011. Ethics approval in our institute covers the use of collected clinical information for clinical and prognostic studies.

**Figure 1:**
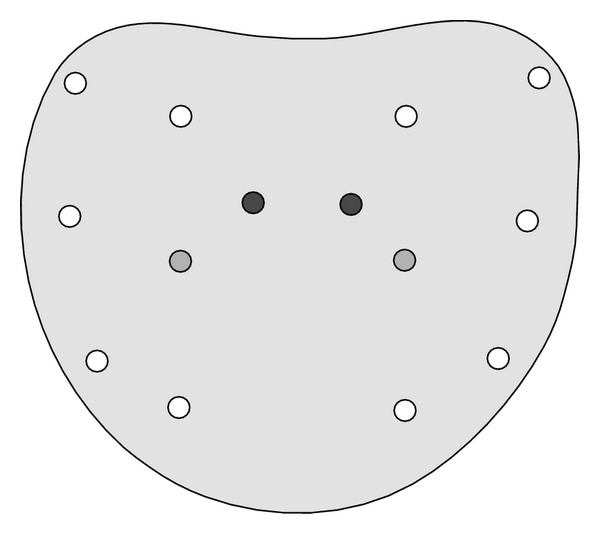
Modified Gore protocol consisting of 2 biopsies of each base, mid, and apex along with routine biopsy of the transitional zone.

 All patients were prescribed prophylactic antibiotics and received a fleet enema at least 12 hours prior to the procedure. The patients were placed in the left lateral decubitus position with bent knees. The transducer probe was covered with a condom and placed in the rectum. All biopsies were performed under local anaesthesia (5 to 10 cc of 2% lidocaine) by a single surgeon (>100 procedures/year and >15 years of experience using TRUS-guided biopsies in his routine practice) using 18 gauge automated spring loaded biopsy gun providing a 22 mm long tissue cores. The systematic biopsy patterns targeted 7 sectors bilaterally: transition zone, apex, center, and base, each medially and laterally, according to the modified Gore protocol shown in Figure 1 as this model provides a greater positive predictive value [12].

 We divided the patients into two groups. The first group consisted of our last 110 consecutive eligible patients who underwent GS 2D TRUS-guided systematic biopsy. The gland was assessed by a transrectal probe at a frequency of 7.5 MHz and scanned from apex to base. The needle was mechanically aligned with the ultrasound image plane via a rigidly attached tubular needle guide which makes it possible to visualize the needle trajectory in the ultrasound images. The second group consisted of our first 110 eligible patients who underwent GS 3D TRUS guided systematic biopsy according to the same protocol. The gland was assessed by an end firing, 3D TRUS probe (3D5-9EK), and a Sonoace X8 ultrasound machine (Medison/koelis urostation) capable of 3D image acquisition allowing real-time 3D TRUS registration system to spatially map each biopsy needle trajectory (Organ Based Tracking). Each biopsy was done by holding the end firing 3D TRUS by the right hand of the operator without an external support; a process called freehand. Initially, a 3D referenced prostate image, named the panorama image, was constructed by integrating 3 sets of 3D TRUS volume data acquired from 3 angles to capture the entire prostate image. Immediately after firing, the needle was left indwelling in the prostate. Real-time 3D TRUS data were acquired during only three seconds and transferred to the workstation. The biopsy tract appeared as a hyperechoic trajectory and the exact spatial coordinates of the proximal and distal ends of each needle trajectory were noted.

 Each biopsy core was fixed separately in 10% formalin and its precise location was recorded. Each biopsy specimen, embedded in paraffin, was serially cut at 3 *μ*m intervals, and subsequently histochemically stained with a freshly made haematoxylin and eosin solution for the microscopy observation by the same uropathologist. For each patient, anatomoclinical parameters, including the location of each core, the number of total and positive cores, the percentage of neoplastic disease, and the Gleason score, were noted. 

 Statistical analysis was done using Stata version 11. We calculated the median of continuous variables and the percentages for categorical ones. Pearson chi-square tests were used to evaluate the association between categorical variables. All reported *P* values are two sided and statistical significance was set at *P* < 0.05.

## 3. Results

Table 1 reports the characteristics of patients and cancer detection rate in group 1 and group 2. No significant difference was noted between patients in group 1 and patients in group 2 in terms of age, DRE, total PSA, and prostate volume. PC detection rate in group 2 was significantly higher than PC detection rate in group 1. A total of 55 out of 110 (50.0%) patients were found positive for PC compared to 37 out of 110 (33.6%) patients in group 1 (*P* < 0.05). In addition, the mean cancer volume detected in group 2 was significantly higher using 3D TRUS-guided prostate sampling (35.1% in group 2 compared to 8.6% in group 1, *P* < 0.05) (Table 2).

 PC grading score was identical in both groups as there was no statistically significant difference in terms of Gleason scores: 19/37 (51.3%) had a Gleason score 6, 14/37 (37.9%) had a Gleason score 7, and 4/37 (10.8%) had a Gleason score >7 in group 1 compared to 31/55 (56.4%) with a Gleason score 6, 19/55 (34.5%) with a Gleason score 7, and 5/55 (9.1%) with a Gleason score >7 in group 2 (*P* > 0.05) (Table 2). 

 In order to determine whether the higher rate of detection of the 3D system is due to the detection of nonsignificant or potentially indolent cancers, a subgroup analysis was done for patients with Gleason score 6 at biopsy, as those having Gleason score ≥7 fall outside the European Association of Urology criteria for active surveillance [12] and therefore were considered to have clinically significant pathologies. Any Gleason score 6 patient with 2 or less positive cores with less than 50% of cancer length in each core was considered to have a potentially nonsignificant cancer. In total, 6 out of 37 patients in group 1 and 15 out of 55 patients in group 2 were found to fulfill the aforementioned criteria. Table 2 reveals that there was no statistically significant difference when comparing the 2 groups for potentially clinically insignificant PC. 

## 4. Comment

Despite a half century of interest and effort to increase the sensitivity and the specificity of prostate biopsy, PC detection is still inadequate at the initial biopsy with reported rates, in the literature, no more than 30–40% in a screened population (33.6% in our series) [4–7].

 A first step to improve PC detection rate was to improve sampling by increasing the number of random biopsy cores and the number of target regions [7, 9, 13–15]. This was still a lottery because 2D TRUS-guided systematic biopsy has the clear limitation of being unable to record the precise 3D location of the needle biopsy tract and TRUS images are only useful for the identification of anatomic landmarks. This leads to inaccurate sampling and mapping of the biopsy cores that are not always homogeneously distributed and tend to be clustered despite attempts at symmetrical placement [16]. A second step was to improve mapping based on a transperineal external grid templates. Even when a transperineal external grid based mapping technique is used, biopsy needle deflection and deformation, anatomical shift of the prostate and periprostatic hemorrhage, and edema occurring during the procedure make precise 3D anatomical spatial distribution of the needle tract in the prostate difficult [17–19]. As such, developments in TRUS technology focused on improving tracking of the actual needle position under real-time 3D and on the ability to accurately retarget the same location. The computer assisted 3D TRUS localization system allows each biopsy to be performed under real time in the real 3D space of the prostate and precisely records the 3D site of each biopsy in the prostate as a reality, and if a none covered area is noted, it is possible to simulate the next biopsy before firing, in order to adequately cover the area of interest. Many case series reported higher cancer detection rates with the computer assisted 3D TRUS localization system [20–25].

To our knowledge, this is the first clinical study comparing, in routine practice, the same systematic biopsy schemes (Gore protocol) performed under 2D or 3D guidance. According to our results, computer assisted 3D TRUS localization system improves PC detection rate in day-to-day practice compared to GS 2D TRUS-guided systematic biopsy done by the same experienced operator in a comparable cohort of patients and using the same biopsy schemes. Higher PC detection rate under 3D biopsy tracking in our series (50% versus 33.6%), comparable to the literature [20–25], is probably related to an improved systematic biopsy due to a wider homogeneous sampling from the built-in sites. Moreover, we found a statistically significant difference in the cancer core rates between the two approaches (35.1% versus 8.6%). A current challenge in PC diagnosis is to identify patients who warrant definitive treatments. All prostate biopsy protocols must be manipulated to detect clinically significant cancer. Many studies have evaluated the detection rate of PC for various biopsy schemes, including the transperineal approach; few have investigated the detection of clinically significant cancers. Our data are even more encouraging when comparing clinically insignificant prostate cancer detection rate between the 2 cohorts and the fact that we compare the last 110 patients of a long experience of 2D TRUS to the first 110 patients of a cohort could be considered as a beginning learning curve. 

**Figure 2:**
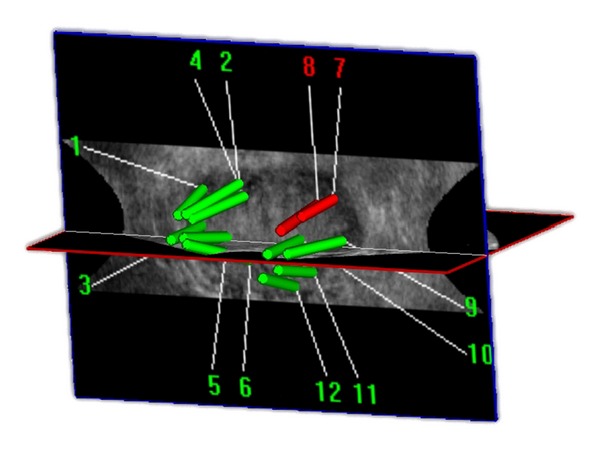
3D trajectory visualization after biopsy along with mapping and cartography.

In daily practice, the biopsy is often performed in many centres by residents and assistants who do not have a long term experience. Although in our study the biopsy was performed by an experienced surgeon, the method is easy to teach while assessing, in real time, quality control. The 3D system is user-friendly, fast, and freehand with no cumbersome additional material required and no need to change usual clinical practices of TRUS biopsy. It uniquely involves analysis of ultrasound images and compensates for intraoperative prostate or patient motion bringing therefore accuracy and hence confidence. Another important point is the labeling of each biopsy which allows for a precise cartography of the prostate (Figure 2). In addition, this novel software allows combining biopsy histological data on the 3D image data, creating a prostate 3D histogram that could facilitate targeted focal therapy. The individual record of the spatial location of previous biopsy specimens would enable a revisiting intervention in case of active surveillance or a rising PSA.

Furthermore, the system allows MRI/TRUS fusion to target the suspicious area delineated on MRI imaging with the potential of improving PC diagnosis and therapy [22, 26–28]. Recently, real-time MRI-guided biopsy technology became clinically available [29]. Considering the high cost and lack of availability of MRI systems and those two million prostate biopsies performed every year in the western countries, MRI-guided prostate biopsy cannot be considered as a routine practice procedure comparable to the 3D system. 

## 5. Conclusions

Nowadays, GS 2D TRUS is the most used imaging modality to guide prostate biopsy. Although additional prospective randomized controlled trials are needed, the current study demonstrates higher detection rate of clinically significant prostate cancer justifying the routine application in clinical practice of a real-time 3D TRUS-guided prostate biopsy system. With the possibility of MRI/TRUS fusion, 3D tracking system is a relevant step toward a reliable prostate cancer diagnostic procedure.

## Figures and Tables

**Table 1:** Patient characteristics and cancer detection rate.

	Group 1	Group 2	*P* value
Number of patients	110	110	
Age (years)	64.2	65.1	0.11
PSA (median, ng/mL)	9.2	10.3	0.13
Prostate volume (median, cc)	47	51	0.09
Cancer detection rate	33.6%	50.0%	<0.05

**Table 2:** Distribution of anatomopathological findings among patients with positive biopsy in each group.

Patient with positive biopsy	Group 1	Group 2	*P* value
Gleason score 6 (%)	19 (51.3)	31 (56.4)	0.23
Gleason score 7 (%)	14 (37.9)	19 (34.5)	0.18
Gleason score >7 (%)	4 (10.8)	5 (9.1)	0.15
Mean percentage of cores involved	8.6	35.1	<0.05
Clinically nonsignificant criteria (%)	6 (16.2)	15 (27.3)	0.2
